# Axillary lymph node dissection versus radiotherapy in breast cancer with positive sentinel nodes after neoadjuvant therapy (ADARNAT trial)

**DOI:** 10.3389/fonc.2023.1184021

**Published:** 2023-08-09

**Authors:** Amparo Garcia-Tejedor, Carlos Ortega-Exposito, Sira Salinas, Ana Luzardo-González, Catalina Falo, Evelyn Martinez-Pérez, Héctor Pérez-Montero, M. Teresa Soler-Monsó, Maria-Teresa Bajen, Ana Benitez, Raul Ortega, Anna Petit, Anna Guma, Miriam Campos, Maria J. Plà, Sonia Pernas, Judith Peñafiel, Carlos Yeste, Miguel Gil-Gil, Ferran Guedea, Jordi Ponce, Maria Laplana

**Affiliations:** ^1^ Department of Gynaecology, Multidisciplinary Breast Cancer Unit, Hospital Universitari Bellvitge, Instituto de Investigación Biomédica de Bellvitge, Barcelona, Spain; ^2^ Rehabilitation Service, Multidisciplinary Breast Cancer Unit, Hospital Universitari Bellvitge, Instituto de Investigación Biomédica de Bellvitge, Barcelona, Spain; ^3^ Department of Medical Oncology, Multidisciplinary Breast Cancer Unit, Institut Català d’Oncología, Instituto de Investigación Biomédica de Bellvitge, Barcelona, Spain; ^4^ Department of Radiation Oncology, Multidisciplinary Breast Cancer Unit. Institut Català d’Oncología, Instituto de Investigación Biomédica de Bellvitge, Barcelona, Spain; ^5^ Department of Pathology, Multidisciplinary Breast Cancer Unit, Hospital Universitari Bellvitge, Instituto de Investigación Biomédica de Bellvitge, Barcelona, Spain; ^6^ Department of Nuclear Medicine, Multidisciplinary Breast Cancer Unit, Hospital Universitari Bellvitge, Instituto de Investigación Biomédica de Bellvitge, Barcelona, Spain; ^7^ Department of Radiology, Multidisciplinary Breast Cancer Unit, Hospital Universitari Bellvitge, Instituto de Investigación Biomédica de Bellvitge, Barcelona, Spain; ^8^ Biostatistics Unit, Instituto de Investigación Biomédica de Bellvitge, Barcelona, Spain; ^9^ Degree in Biology, Monitoring, Instituto de Investigación Biomédica de Bellvitge, Barcelona, Spain

**Keywords:** axillary dissection, axillary radiotherapy, neoadjuvant systemic therapy, breast cancer, sentinel lymph node metastases

## Abstract

**Introduction:**

Breast cancer surgery currently focuses on de-escalating treatment without compromising patient survival. Axillary radiotherapy (ART) now replaces axillary lymph node dissection (ALND) in patients with limited sentinel lymph node (SLN) involvement during the primary surgery, and this has significantly reduced the incidence of lymphedema without worsening the prognosis. However, patients treated with neoadjuvant systemic treatment (NST) cannot benefit from this option despite the low incidence of residual disease in the armpit in most cases. Data regarding the use of radiotherapy instead of ALND in this population are lacking. This study will assess whether ART is non-inferior to ALND in terms of recurrence and overall survival in patients with positive SLN after NST, including whether it reduces surgery-related adverse effects.

**Methods and analyses:**

This multicenter, randomized, open-label, phase 3 trial will enroll 1660 patients with breast cancer and positive SLNs following NST in approximately 50 Spanish centers over 3 years. Patients will be stratified by NST regimen and nodal involvement (isolated tumoral cells or micrometastasis versus macrometastasis) and randomly assigned 1:1 to ART without ALND (study arm) or ALND alone (control arm). Level 3 and supraclavicular radiotherapy will be added in both arms. The primary outcome is the 5-year axillary recurrence determined by clinical and radiological examination. The secondary outcomes include lymphedema or arm dysfunction, quality of life based (EORTC QLQ-C30 and QLQ-BR23 questionnaires), disease-free survival, and overall survival.

**Discussion:**

This study aims to provide data to confirm the efficacy and safety of ART over ALND in patients with a positive SLN after NST, together with the impact on morbidity.

**Ethics and dissemination:**

The Research Ethics Committee of Bellvitge University Hospital approved this trial (Protocol Record PR148/21, version 3, 1/2/2022) and all patients must provide written informed consent. The involvement of around 50 centers across Spain will facilitate the dissemination of our results.

**Trial registration:**

ClinicalTrials.gov, identifier number NCT04889924.

## Strengths and limitations of this study

1. The axillary Dissection versus Axillary Radiotherapy after Neoadjuvant Therapy (ADARNAT) study is one of only a few phase 3 clinical trials to assess axillary management in patients with limited positive sentinel lymph nodes (SLNs) after neoadjuvant therapy, including chemotherapy or endocrine therapy.

2. The size of the study, including 1660 patients with breast cancer, is adequate to confirm the hypothesis that all patients with ≤2 positive SLNs after neoadjuvant treatment may avoid complete axillary lymph node dissection if treated with axillary radiotherapy.

3. The multicenter design that includes around 50 hospitals across Spain will ensure the reproducibility of the results and will facilitate their dissemination.

4. An important limitation will be the involvement of a large number of hospitals, with different SLN techniques and different professionals at both diagnosis and surgery. However, the randomization has been performed independently in each hospital, so this bias should be solved.

5. Another important limitation will be the low event rate (i.e., axillary recurrence is approximately 2% at 5 years).

## Introduction

The current trend in breast cancer treatment focuses on de-escalation without compromising patient survival to improve quality of life and reduce complications. Axillary lymph node status is an important prognostic factor that may contribute to decisions about adjuvant therapy. Although axillary lymph node dissection (ALND) allows us to achieve locoregional disease control, it causes adverse functional sequelae, including lymphedema and restricted shoulder mobility, in a non-negligible percentage of patients (1, 2). Furthermore, the contribution of ALND to breast cancer survival is controversial, leading to selective sentinel lymph node biopsy (SLNB) becoming the standard approach in patients with invasive breast cancer and no lymph node involvement at diagnosis (cN0). However, ALND has remained the standard of care for patients with positive SLNB.

Since the publication of the ACOSOG-Z0011 (3), AMAROS (4) and IBCSG 23-01 (5) trials, it has been possible to de-escalate surgery further by omitting lymphadenectomy in those with only one or two SLNs at the primary surgery. Irradiation in the lymph node areas has been shown to have the same efficacy but reduces the morbidity of ALND (6, 7) while maintaining good local control of axillary disease without worsening the prognosis. However, this has not been evaluated in patients receiving neoadjuvant systemic therapy (NST) and have limited SLN involvement. NST includes both chemotherapy (CT) and endocrine therapy (ET). ALND remains the standard procedure in this group if they have residual lymphatic involvement in the SLNB (ypN+). Thereafter, most cases receive lymph node irradiation, increasing the risk of lymphedema due to the combination of axillary surgery and radiotherapy (RT) (8, 9).

Current NST regimens achieve a high percentage of pathological complete response (pCR) at both the mammary and axillary levels: 60%–80% in HER2-positive disease, depending on hormone receptor (HR) status; 40%–60% in triple-negatives; and 21%–30% in luminal B HER2 (10–14). However, no published data exist in support of not performing ALND in patients with residual lymph node disease after NST, when axillary radiotherapy is performed. The Mayo Clinic’s ALLIANCE A11202 multicenter study is ongoing (https://clinicaltrials.gov/ct2/show/NCT01901094), aiming to compare ALND and axillary RT (ART) in node-positive breast cancer after CT, though it does not consider ET. Advances in systemic therapy have increased the probability of achieving pCR. By avoiding axillary surgery, offsetting treatment with nodal irradiation, we can prevent unnecessary morbidity. Given the current uncertainties with primary surgery, research must consider whether ART can replace ALND in patients with limited SLN involvement after NST (CT or ET). To be considered effective, this must also maintain current locoregional control and survival rates, together with the incidence of treatment-related adverse effects. The Axillary Dissection versus Axillary Radiotherapy and Neoadjuvant Therapy (ADARNAT) study aims to resolve these uncertainties.

## Objective

The primary objective of the ADARNAT trial is to evaluate whether ART is non-inferior to ALND in terms of 5-year axillary recurrence. Secondary objectives include assessment of whether ART reduces surgery-related adverse effects and improves quality of life, disease-free survival (DFS), and overall survival (OS).

## Methods and analysis

### Study design

The ADARNAT trial is a randomized, open-label, multicenter, phase 3 non-inferiority trial. It will enroll 1660 patients with breast cancer and positive SLNB who receive NST from 50 centers across Spain. It will be conducted over a 3-year period with 5 years’ follow-up. A preliminary study will be carried out with 820 patients over 3 years to evaluate the secondary objective on adverse effects related to surgery.

**Figure 1:**
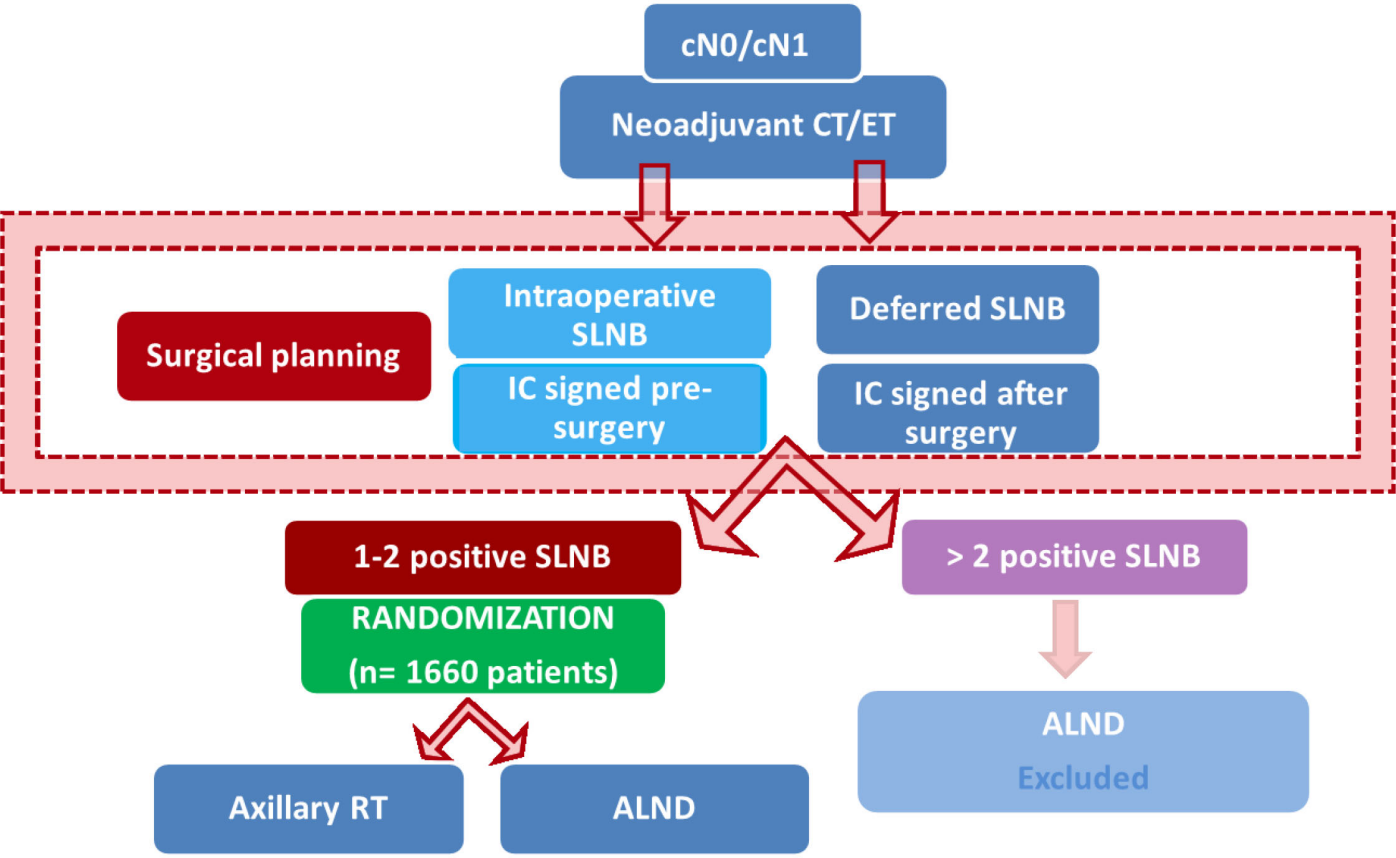
Schedule. CT, Chemotherapy; ET, Endocrine Therapy; SLNB, Sentinel Lymph Node Biopsy; IC, Informed Consent; RT, Radiotherapy; ALND, Axillary Lymph Node Dissection.

**Figure 2:**
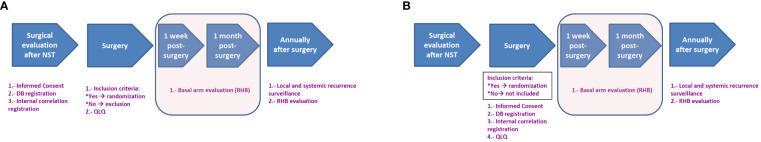
Chronogram. NST, Neoadjuvant Systemic Therapy; DB, Data Base; QLQ, Quality of Life Questionnaire; RHB, Rehabilitation service. **(A)** Chronogram when intraoperative SLNB. **(B)** Chronogram when postoperative SLNB result.

Patients will be stratified by NST regimen (CT or ET) and SLN involvement (isolated tumoral cells/micrometastases or macrometastases) and will be randomly assigned (1:1) to ART without ALND (study arm) or ALND alone (control arm). **Table 1** and **Figure 1** show the enrolment, randomization, and intervention schedules, while **Figure 2** shows the chronogram. The coordinating centers are the Hospital Universitario Bellvitge & Institut Catala d’Oncologia, in charge of the dissemination, implementation and monitoring of the study in all the centers (see author’s contribution). The SPIRIT Checklist, 2013, is completed in **Additional File 1**.

**Table 1:** Work plan.

			2022			2023-2024			2025			2026-27			2028		
Task	Person responsible	Status	S1	S2	Q3	Q1	Q2	Q3	Q1	Q2	Q3	Q1	Q2	Q3	Q1	Q2	Q3
**Pilot study**																	
Institutional review board	Main PI	Complete															
ClinicalTrials.gov Identifier	Main PI	Complete															
Protocol design and electronic database	Main PI	Complete															
Multicenter review board, contracts	All PI	In progress															
Multicenter protocol implementation	Main PI	In progress															
Coordination	Main PI	In progress															
Recruitment and randomization	All PI	Complete															
Treatment	S & R	Complete															
Follow-up	O & R & Rh	In progress															
Fill in the sheet	All PI	In progress															
Monitoring	Monitor	In progress															
Feasibility analysis	Main PI	Not started															
Statistical analyses	Statistic	Not started															
Publication	Main PI	Not started															
**Main study**																	
Coordination	Main PI	In progress															
Recruitment and randomization	All PI	In progress															
Treatment	S & R	In progress															
Follow-up	O & R & Rh	Not started															
Fill in the sheet	Data manager	Not started															
Monitoring	Monitor	Not started															
Statistical analyses	Statistic	Not started															
Publication	Main PI	Not started															

PI, Principal Investigator; S & R, Surgeons and Radiation Oncologist; O & R & Rh, Medical Oncologists, Radiation Oncologist and Rehabilitation Service.

The gray shadow represents the foreseeable months for the pilot study and the black shadow for the main study.

### Sample size

The sample size was calculated by considering the design of the 2014 EORTC-AMAROS study (3). This used a similar primary outcome to ours, except that all their patients were within the primary surgery setting and ours had undergone NST. We assume a 5-year axillary recurrence of 2% in the group with lymph node dissection. The limit of non-inferiority of the hazard ratio of relapses between control and treatment is 2, implying a maximum axillary recurrence rate of approximately 4% in the intervention group at 5 years. If we expect the hazard ratio of axillary recurrence between control and treatment to be 1, the study will require 2571 subjects per to reject the null hypothesis of non-inferiority; that is, to observe 52 events, accepting a type I risk of 2.5% and a power of 80%. Due to the low incidence in the 2014 EORTC-AMAROS study, they reported that the estimated number of events would probably never occur. Therefore, the ethics committee allowed them to finalize the analysis with 441 patients, reducing the power of the primary endpoint of non-inferiority. Nevertheless, their results indicated comparable regional control at 5 years in both groups. In the other multicenter study like ours, *coordinated through the Alliance A 11202 led by a Mayo Clinic PI*, the estimated sample size was 1660 patients for assessing non-inferiority and DFS at 5 years. Given that one of our objectives is to maintain equivalent DFS at 5 years in both treatment arms while reducing comorbidity due to axillary radiotherapy, we opted to use the same sample size of 1660 patients.

A pilot study is planned after we have included 12% of the sample (200 patients), to assess the feasibility and safety of this study. Moreover, a preliminary study to assess the adverse effects associated with surgery, taking into account that the proportion of lymphedema is expected to be around 15% in the radiotherapy group and 28% in the lymphadenectomy group, accepting an alpha risk of 0.05 and a beta risk less than 0.2 in a bilateral contrast, 205 subjects are needed in the first group and 205 in the second to detect the difference between two proportions as statistically significant, which means a total of 410 patients. A rate of loss to follow-up of 25% has been estimated. The ARCSINUS approximation has been used.

Since the biological and prognostic behavior is very different between the tumors for which neoadjuvant chemotherapy is indicated and those for neoadjuvant endocrine therapy, it is expected to have an identical sample of patients in each of the treatment groups to facilitate the analysis and to be able to extrapolate the results to the general population, so the preliminary study will be carried out with 820 patients (410 after endocrine treatment and 410 after chemotherapy).

### Recruitment

Patients meeting the inclusion criteria will be enrolled at the following centers: Bellvitge University Hospital-Catalan Institute of Oncology-L’Hospitalet (Barcelona), Viladecans Hospital (Barcelona), Parc Taulí Hospital (Barcelona), Mutua de Terrassa (Barcelona), Germans Trias i Pujol Hospital (Barcelona), Consorci Sanitari of Terrasa (Barcelona); Althaia Fundation of Manresa (Barcelona), Hospital del Mar (Barcelona); Consorci Sanitari Integral Moisès Broggi (Barcelona), Clinical and Provincial Hospital of Barcelona (Barcelona); Alvaro Cunqueiro Hospital (Vigo); Cruces University Hospital (Vigo); Ribera Povisa Hospital (Pontevedra); Montecelo Hospital (Pontevedra); Valencian Institute of Oncology (Valencia); Central University Hospital of Asturias (Principality of Asturias); Infanta Sofía University Hospital (Madrid), Ramon y Cajal University Hospital (Madrid), San Chinarro University Hospital (Madrid), La Paz University Hospital (Madrid), Puerta del Hierro University Hospital (Madrid), University Hospital 12 de Octubre (Madrid); Gregorio Marañón General University Hospital (Madrid); Recoletas Campo Grande Hospital (Valladolid); Clinic University Hospital of Valladolid (Valladolid); Donostia University Hospital (Guipúzcoa); Virgen Macarena (Sevilla); Virgen de las Nieves Hospital (Granada); San Cecilio University Hospital (Granada); Burgos University Hospital (Burgos); Virgen del Camino Hospital (Pamplona); Virgen de la Arrixaca Hospital (Murcia), A Coruña University Hospital (La Coruña); San Cristobal de la Laguna University Hospital Santa Cruz de Tenerife; Nuestra Señora de la Candelaria Hospital (Santa Cruz de Tenerife); Trueta Hospital and Catalan Institute of Oncology (Girona); Alava University Hospital (Vitoria); Virgen de la Victoria University Hospital (Malaga) and Regional University Hospital (Malaga); Puerta del Mar University Hospital (Cádiz); Joan XXIII University Hospital (Tarragona); Sant Joan de Reus University Hospital (Tarragona), and Galdakao-Usansolo University Hospital (Vizcaya).

### Eligibility criteria

**Figure 3:**
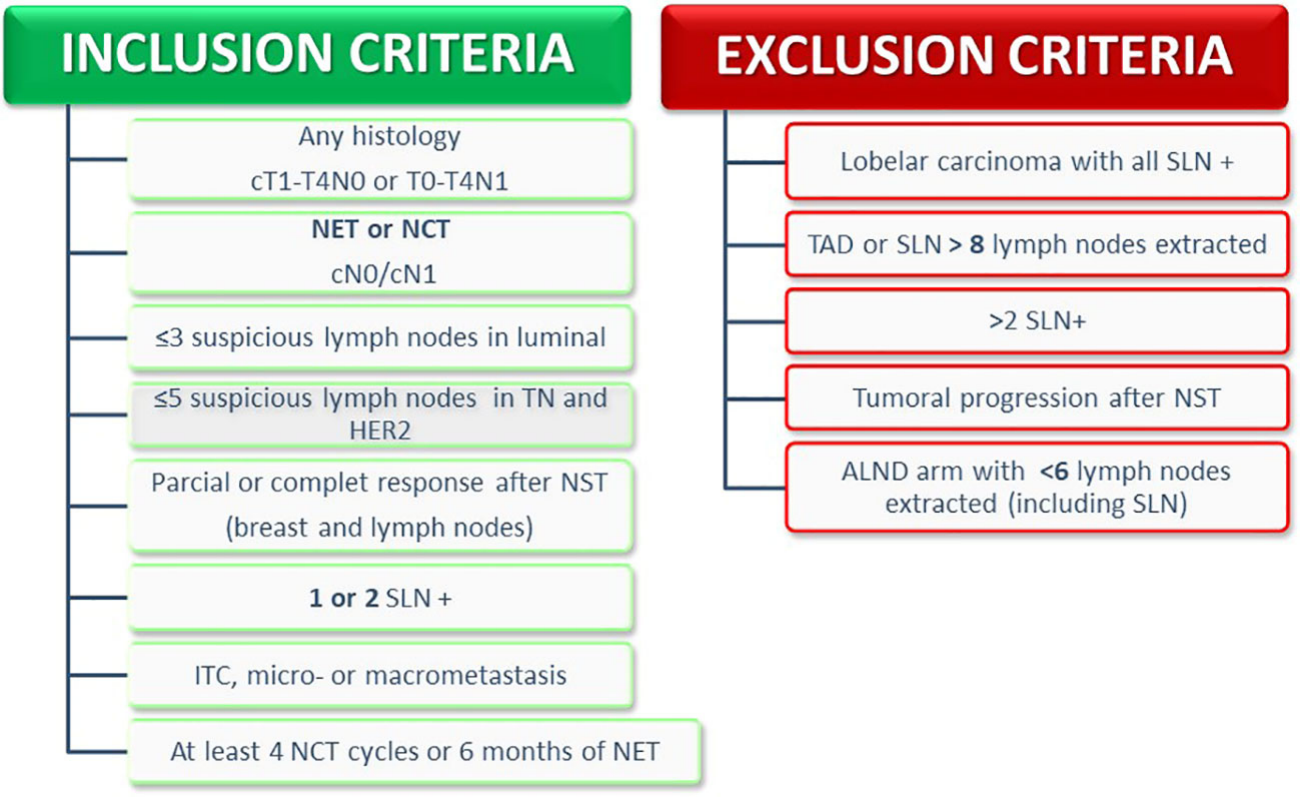
Inclusion and exclusion criteria. NCT, Neoadjuvant Chemotherapy; NET, Neoadjuvant Endocrine Therapy; SLNB, Sentinel Lymph Node Biopsy; TAD, Target Axillary Dissection; ALND, Axillary Lymph Node Dissection.

The inclusion and exclusion criteria are summarized in **Figure 3**.

#### Inclusion criteria

Patients are eligible for inclusion if they meet all the following criteria:

(1) Female or male, age ≥18 years.
(2) Performance status: Karnofsky ≥70% and Barthel ≥60 points.
(3) Patients suitable for SLN or target axillary dissection (TAD) after neoadjuvant treatment (ET or CT), with clinical stage T1-T4b N0 or Tx-T4b N1 at diagnosis. Histologically confirmed invasive breast cancer with estrogen receptor, progesterone receptor, Ki-67, and HER-2 status assessed before starting NST.
(4) Axillary ultrasound at diagnosis with ≤3 suspicious lymph nodes in luminal tumors and ≤5 suspicious lymph nodes in triple-negative and HER2-positive tumors.
(5) Axillary images after NST with signs of response (complete imaging response is not necessary).
(6) In cN1 cases before NST, a minimum of 3 SLN should be removed if none have been marked. In both cases, less than 8 lymph nodes should be removed, including the SLN and non-sentinel nodes.
(7) 1 or 2 lymph nodes involved (micrometastasis and macrometastasis) in the definitive anatomopathological study of the SLN or TAD.
(8) In case of cN0 before NST, all patients with ≤2 affected SLNs will be included, regardless of the number of lymph nodes removed.
(9) To complete at least 4 cycles of neoadjuvant CT or 6 months of ET.

#### Exclusion criteria

Patients are excluded if they have any of the following:

(1) cT4d and/or cN2.
(2) ypN0.
(3) Positive extra-axillary sentinel node (intramammary or internal mammary) as the only node affected if the axillary SLN is negative, since axillary lymphadenectomy would not be justified.
(4) Axillary lymphadenectomy for <6 lymph nodes, including those of the TAD or SLN.
(5) Distant metastasis at diagnosis.
(6) Progression during NST.
(7) Participation in another clinical trial whose endpoint was local axillary recurrence.
(8) Neoadjuvant RT.
(9) History of breast surgery for ipsilateral cancer in the past 10 years.
(10) History of previous axillary surgery.
(11) History of other cancer in the last 5 years, except squamous cell carcinoma of the skin or basal cell carcinoma.
(12) Bilateral tumors when the contralateral tumor has a worse prognosis.
(13) Previous breast or thoracic irradiation for any reason (e.g., lung, lymphoma, etc.).
(14) Pregnancy.

### Randomization and arms

After signing an informed consent form, eligible participants will be included by the surgeon *via* a secure online platform (REDCAP), stratified by NST and SLN involvement, and randomly allocated to the study or control arm in a 1:1 ratio: (1) study arm: Axillary RT without lymphadenectomy; and (2) control arm: axillary lymphadenectomy. Both arms will complete level 3 local axillary treatment with supraclavicular irradiation with/without the internal mammary chain (as indicated).

### Interventions

**1. Primary histological diagnosis by core needle biopsy.** Each pathological anatomy department will perform conventional techniques with the following recommendations: (a) study of prognostic factors using American Society of Clinical Oncology (ASCO) guidelines; (b) surrogate molecular classification using several prognostic factors (15): luminal A-like (estrogen receptor [ER] >10% + progesterone receptor [PR] >20% + Ki-67 <20% + HER2 negative), luminal B-like HER2-negative (ER >10% + HER2-negative + RP <10% and/or Ki-67 >20%), luminal B-like HER2 + (HR >10% + HER2 positive), HER2 positive (HER2 positive + HR <10%), triple-negative (HR<10% + HER2 negative).

**2. Pre-NST axillary assessment.** An axillary ultrasound will be performed on all patients before starting NST. Cortical thickness will be measured, applying the Bedi criteria (16) based on cortical morphology (suspicious if Bedi ≥3). The number of suspicious nodes will be indicated by axillary ultrasound, and if possible, MRI. Fine needle aspiration or core needle biopsy should be performed for histology. It is recommended to mark 1 pre-NST pathological lymph node with a clip or seed (preferably the most suspicious).

**3. Treatment with NST.** (1) Anthracycline- and taxane-based CT. Patients with HER2-positive disease will be treated with CT plus dual HER2 blockade with trastuzumab and pertuzumab. In patients aged <65 years with triple-negative breast cancer, carboplatin will be used in combination with paclitaxel, followed by anthracyclines. In case of toxicity requiring treatment suspension, those patients who have completed at least 3 months of treatment may be included. (b) ET: aromatase inhibitor (letrozole 2.5 mg, anastrozole 1 mg, or exemestane 25 mg daily for 6–12 months in postmenopausal women ± CDK4/6 inhibitor, or Tamoxifen 20 mg daily for 6–12 months in men. ET was indicated for postmenopausal patients, cN0 at diagnosis with tumors that express hormone receptors (ER and/or PR) >40%, Ki-67 <25%, and that are HER2 negative.

**4. Assessment of NST response.** Response evaluations will be assessed by clinical examination, mammography-ultrasound, and MRI before and after NST. The World Health Organization criteria (17) will be applied for categorization into 4 categories: (1) complete response; (2) partial response; (3) stability; and (4) progression (to be excluded).

**5. Breast and axillary surgery.** Surgery on the breast and axilla should be performed 3–6 weeks after finishing neoadjuvant CT and 6–12 months after starting ET. Breast surgery may be conservative or mastectomy with or without associated oncoplastic techniques, depending on the clinical indication. The axillary approach will depend on the pre-NST lymph node status: (a) cN0 pre-NST, the conventional SLN technique will be performed with radiocolloid, patent blue, or any other technique validated by the center; and (b) cN1 pre-NST, with SLN excision using a dual technique, indocyanine green marking, TAD, or a technique validated in a given center. A maximum of 2 affected SLNs will be accepted for study inclusion.

**6. Anatomical-pathological study of the SLN or TAD.** This will be performed by histological study or one step nucleic acid amplification (OSNA), according to the usual technique in each center. Micrometastasis will be considered when the histological size is >0.2 but <2 mm or when OSNA reveals >250 but <5000 copies. Macrometastasis will be considered when the histological size is >2 mm or OSNA reveals >5000 copies.

**7. Local treatment after randomization will depend on the cohort.**

**a.** Study arm: axillary RT without lymphadenectomy: RT will be administered to the affected breast/chest wall and all lymph node chains, including Berg levels 1–3 and the supraclavicular chain with/without the internal mammary chain, according to each hospital’s protocol. It will start 3–12 weeks after surgery (except cases receiving adjuvant CT after surgery, when postponement to 3 weeks after finishing CT is permissible) using any available planning technique: 3-dimensional conformal RT (3D-CRT), intensity-modulated RT (including volumetric-modulated arc radiotherapy), or proton radiation therapy. The dose and fractionation schedules accepted in the clinical trial protocol are as follows: normofractionation of 50 Gy administered over 25 fractions, at 2 Gy/fraction, 5 days a week; or moderate hypofractionation of 40.05 Gy administered over 15 fractions at 2.67 Gy/fraction, 5 days a week (the START scheme). If a tumor bed booster dose is recommended, the prescribed dose can be 10–20 Gy at 2 Gy/fraction or a radiobiological equivalent to 100% of the isodose, treated once daily. A boost should follow full breast radiation therapy either without a break or simultaneously.

**b.** Control arm: axillary lymphadenectomy: intraoperative or deleted ALND will be allowed. Before 4 weeks after the previous surgery, axillary lymphadenectomy will be completed including Berg levels I–II, removing a minimum of 6 or 10 lymph nodes (TAD + lymphadenectomy). It is not necessary to complete Berg level 3 dissection, but it can be included at the discretion of the surgeon if involvement is suspected. If ≤6 nodes are removed, the patient will be excluded from the study. Breast/chest wall RT and lymph node chains corresponding to level 3 and supraclavicular with/without the internal mammary chain will also be irradiated on the same basis, as approved by international guidelines. In this cohort, we will also include Berg levels I–II in the irradiation field when extranodal tissue is affected, either by invasion of the perinodal fat or capsular rupture.

**8. Anatomical-pathological exam of the surgical specimen.** The diameter of the tumor bed and the percentage of residual cellularity will be considered to calculate the residual cancer burden (RCB) according to Symmans criteria: RCB 0 (no breast or lymph node carcinoma), RCB 1 and 2 (partial response), and RCB 3 (chemoresistance). The post-NST response can also be assessed with the Miller and Pain criteria (response divided into 5 grades by cellularity before and after neoadjuvant therapy, without considering axillary metastasis or vascular invasion). In patients treated with ET, the Preoperative Endocrine Prognostic Index (PEPI) score will be applied by tumor size, lymph node involvement, %ER, and Ki-67 level.

**9. Adjuvant systemic treatment.** Depending on tumor type, the following recommendations apply: (1) HER2-positive tumors require anti-HER2 treatment with 14 cycles of T-DM1; (2) triple-negative breast cancers require 6–8 cycles of adjuvant capecitabine after radiotherapy; (3) luminal cancers in premenopausal patients require ovarian suppression with luteinizing hormone-releasing analogs and an aromatase inhibitor, while postmenopausal patients required adjuvant treatment with an aromatase inhibitor for 5–10 years depending on tolerance; (4) adjuvant treatment in patients treated with ET will depend on the PEPI score (for PEPI 3, the decision is at the discretion of the investigator and patient, but completing 5–10 years of adjuvant ET is recommended, while for PEPI ≥4, adjuvant CT is assessed if the patient’s conditions allows, followed by ET for 5–10 years. Oncogeriatric assessments are recommended for patients aged >75 years.

**10. Follow-up.** After finishing local treatment, patients will be followed by clinical evaluation every 6 months for the first year, and annual mammography for the first 5 years, the same as the usual oncological follow-up.

**11. Monitoring.** Each center will be monitored twice a year to verify the value of the data and any other possible deviation from the protocol. The monitor independently review all events and thoroughly investigate those adverse events considered serious and unexpected, in parallel with the research team.

### Outcomes

The primary outcome is axillary locoregional recurrence based on imaging tests and confirmed histologically by core needle biopsy or fine needle aspiration. No differences are expected between the study groups.

Secondary outcomes include DFS, OS, quality of life, and local treatment-related adverse effects (lymphedema and shoulder arc limitation). After pre-surgical evaluation, the rehabilitation service should offer follow-up by 1 month after surgery (control arm) or RT (study arm), and annually thereafter for 5 years to exclude collateral adverse effects. Specifically, the secondary outcomes are defined as follows:

- **DFS:** the time from the date of randomization to the date of disease progression (local, regional, or distant recurrence) or death, whichever occurs first.
- **OS:** the time from the date of randomization to the date of death from any cause.
- **Quality of life:** patients will complete the EORTC QLQ-C30 and QLQ-BR23 quality of life questionnaires at baseline and each year, *via* an online platform, to compare the test scales over time. A linear model will be made with a scale from 0 to 100, with differences of >10 points considered clinically relevant.
- **Lymphedema**: the circumference of the healthy and affected arms will be measured with a tape measure, in centimeters, at six anatomical sites: base of the head of the third metacarpal, the wrist at the level of the ulnar styloid, 14 and 7 cm distal to the olecranon, 10 and 20 cm proximal to the olecranon with the elbow flexed at 45° and the shoulder in anatomical position. The excess volume in the affected upper limb will be calculated indirectly using the truncated cone formula. A diagnosis of lymphedema will be considered when the difference in excess volume between both arms exceeds 10%. Based on excess volume, lymphedema severity will be graded as mild (<20%), moderate (20%–40%), or severe (>40%). All patients with a ≥10% excess lymphedema volume received treatment of exercise therapy and skin care, decongestive lymphatic therapy, pressotherapy, and a multilayer bandage during the first phase, before moving on to compression garments in the maintenance phase, in accordance with the international consensus (18).
- **Shoulder arc limitation:** the mobility in flexion, abduction, and external and internal rotation of the affected side is measured and compared with the pre-surgical evaluation in same shoulder, using a goniometer for all measurements. Shoulder mobility will be considered limited when there is a difference of at least 10° in any measure.
- **Other complications:** winged scapula and axillary web syndrome will be assessed. The latter refers to one or more fibrous cords that are palpable and visible in the axillary region and can extend to the wrist).

### Statistical analyses

The demographic and clinical profile of all enrolled subjects will be described by treatment group. Descriptive summaries will be provided for the primary and secondary variables, as appropriate. For the primary endpoint, Kaplan–Meyer curves will be plotted for time to axillary recurrence and compared using the log-rank test. Survival will be estimated at 5 years in each study group with 95% confidence intervals. Graphs will be used to illustrate the results of statistical analyses. We plan to conduct all tests on a 2-sided basis with a significance level of 0.05 and relevant 95% confidence intervals. Further details will be provided in the statistical analysis plan. We plant to use R version 4.1.0 or higher (R Core Team, Vienna, Austria; https://www.R-project.org/) for the statistical analyses.

## Ethics and dissemination

This protocol complies with both the standards of Good Clinical Practice and the ethical principles for clinical research established in the Declaration of Helsinki. All records that identify participants will be kept confidential and, to the extent permitted by applicable laws and/or regulations, will not be made available to the public. We will comply with the provisions for Organic Law 3/2018 concerning the protection of personal data and guarantee of digital rights, and with European Union Regulation 2016/679 concerning data protection. Each patient registered in the study will automatically receive a sequential numerical identification, and their name will not be requested or registered in the database. Data will be included in a database, managed through the REDCAP platform, with access granted only to the research team, monitors, research ethics committees, and regulatory authorities. In case of data transmission to countries outside the European Union or European Economic Area, the promoter guarantees that participant data will be protected by safeguards (e.g., contracts or other mechanisms deemed necessary by data protection authorities).

This project has been registered at ClinicalTrials.gov (NCT04889924) and was approved by the Ethics Committee of the Bellvitge University Hospital (Protocol Record PR148/21). Once the trial is finished, the results will be published regardless of the result. New hospitals are still being activated. A pilot study is currently ongoing for the first 200 enrolled patients and is expected to complete in 2024, with the full study expected to complete in 2028. Both studies will be published once the results have been analyzed.

Any amendment of the protocol will be updated on ClinicalTrials.gov.

We anticipated that the multicenter study design, with the involvement of approximately 50 centers throughout Spain, will facilitate the easy diffusion of results. After completing the full sample follow-up, we plan to present our results at prestigious congresses and to published the results in a high-impact journal (Q1). If our hypothesis is confirmed, many patients will be able to benefit from avoiding axillary lymphadenectomy.

## Ethics statement

The studies involving human participants were reviewed and approved by Research Ethics Committee of Bellvitge University Hospital (Protocol Record PR148/21, version 3, 1/2/2022. The patients/participants provided their written informed consent to participate in this study.

## Author contributions

Conceptualization and overall design: AG-T, JPo and ML. Sample size calculation: JPe, and AG-T. Statistical planning and database platform: JPe. Pathological study design: MS-M and AP. Radiological study design: AG and RO. Axillary approach design: MB and AB. Surgical design: AG-T, CO-E, MC, and MP. Radiotherapeutic design: ML and EM-P. Oncological design: CF and SP. Comorbidity design: SS and AL-G. Monitoring: CY.

## Glossary

**Table:**

ADARNAT	Axillary Dissection versus Axillary Radiotherapy and Neoadjuvant Therapy
ALND	Axillary lymph node dissection
DFS	Disease-free survival
ER	Estrogen receptor
ET	Endocrine therapy
GCP	Good Clinical Practice
HR	Hazard ratio
NST	Neoadjuvant systemic treatment
OS	Overall survival
OSNA	One step nucleic acid amplification
PEPI	Preoperative Endocrine Prognostic Index
PR	Progesterone receptor
RCB	Residual cancer burden
SLN	Sentinel lymph node
SLNB	Sentinel lymph node biopsy
TAD	Target axillary dissection
